# N, O and P co-doped honeycomb-like hierarchical porous carbon derived from *Sophora japonica* for high performance supercapacitors†

**DOI:** 10.1039/c9ra06934h

**Published:** 2019-11-13

**Authors:** Li Zhang, Yuxia Zhu, Guangzhen Zhao, Yanjiang Li, Guang Zhu

**Affiliations:** Key Laboratory of Spin Electron and Nanomaterials of Anhui Higher Education Institutes, Suzhou University Suzhou 234000 PR China zhlisuzh@163.com guangzhu@ahszu.edu.cn

## Abstract

Novel N, O and P co-doped honeycomb-like hierarchically porous carbon (N-O-P-HHPC) materials with a large specific surface area from *Sophora japonica* were prepared *via* a one-step activation and carbonization method and used as an electrode for supercapacitors. The results indicate that as-prepared N-P-HHPC with a large specific surface area (2068.9 m^2^ g^−1^) and N (1.5 atomic%), O (8.4 atomic%) and P (0.4 atomic%) co-doping has a high specific capacitance of 386 F g^−1^ at 1 A g^−1^. Moreover, a 1.8 V symmetrical SC was assembled from the N-O-P-HHPC-3 electrode using 1 M Na_2_SO_4_ gel electrolyte, presenting a high energy density (28.4 W h kg^−1^ at 449.9 W kg^−1^) and a long life cycling stability with only 7.3% capacitance loss after 10 000 cycles. Furthermore, the coin-type symmetrical SC using EMIMBF4 as electrolyte presents an ultrahigh energy density (80.8 W h kg^−1^ at 1500 W kg^−1^). When the two coin-type symmetrical SCs are connected in series, eight red light-emitting diodes (LEDs) and a small display screen can be powered. These results demonstrate as-prepared N, O and P co-doped HHPC is a considerable candidate as a carbon electrode for energy storage devices.

## Abstract 2

N, O and P co-doped honeycomb-like hierarchical porous carbon (N-P-HHPC-3) derived from *Sophora japonica* displays an ultrahigh energy density (80.8 W h kg^−1^ at 1500 W kg^−1^) and outstanding long-term stability.

## Graphical abstract

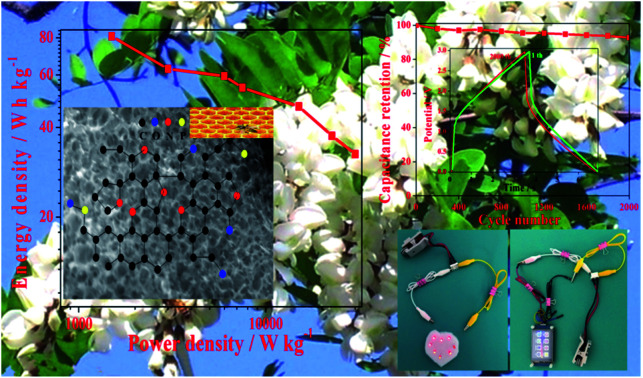

## 1. Introduction

Along with the fast development of the global economy, the energy crisis and environmental pollution caused by the large consumption of non-renewable resources such as coal and oil, have become the main obstacles of economic development.^1–3^ Meanwhile, the development of electron devices and electric vehicles is an indispensable part of economic development. Therefore, developing efficient, green and stable energy storage devices is an important way to solve economic and environmental issues.^4–8^ Supercapacitors (SCs), also named as electrochemical capacitors, have received interest for portable and miniaturized electronics, electric vehicles, *etc.*, due to their ultra-high power density, light weight, fast charge/discharge, outstanding stability and safety and so on.^9,10^ The electrode material as one of the most essential parts affects the electrochemical properties of SCs.

As we know, the charge storage of the carbon derived electrode material for SCs is that charge gathered on the surface to adsorb electrolyte ions.^11–16^ Over a long time, there are some strategies to enhance the electrochemical properties of carbon material, such as porous structure optimization, heteroatom doping and so on.^17–20^ It has been found that a reasonable interconnected porous structure with micropores, mesopores and macropores, which can short the transmission path of ion electrolyte and provide enough adsorption sites for ion electrolyte, and thus can enhance the electrochemical performance.^21,22^ Therefore, carbon material with hierarchical porous nanostructure has became the focus of scholars' attention. On the other hand, the heteroatom (N,^23^ P,^24^ S,^25^ B^26^ and O^21^) doped into carbon material is an effective strategy to improve performance. It has been proved that the heteroatom doping can enhance the conductivity and wettability, and produce pseudocapacitance. Meanwhile, the multi-heteroatoms doping can further improve the capacitance, compared to the one-type-only heteroatom doping.^22,27^

Biomass derived hierarchical porous carbon materials have aroused attention of scholars, owning to their advantages of environmental friendship, low cost and easy access.^28,29^ The most importantly, biomass based hierarchical porous carbon material can inherit nature unique microstructures and the chemical compositions, which can easily produce porous structure and multi-heteroatoms doping.^30,31^ To date, many hierarchical porous framework structures or multi-heteroatoms co-doped carbon materials have been prepared from biomass, such as laozao,^32^ soybeans,^33^*Perilla frutescens*,^34^ rice husk.^35^ During the preparation process, KOH as a activating agent is widely used to etch the carbon matrix leaving porous framework structures (microporous and mesoporous) to create very high surface area.^12,36^ For instance, the specific capacitance of porous carbon derived from fungus with KOH chemical activation is 374 F g^−1^ at current density of 0.5 A g^−1^ than primeval carbon (116 F g^−1^).^37^ Biomass waste based activated carbon material displays a higher specific capacitance (222 F g^−1^ at 1 A g^−1^).^38^ Recently, Xu *et al.* reported that N, S co-doped carbon derived from *Sophora japonica* (SJ) was prepared by a two-step heat treatment process for zinc–air battery.^39^ Despite the above progress to date, the hierarchically porous carbon prepared by SJ used as SCs electrodes has been rarely reported.

In this study, N, O and P co-doped honeycomb-like hierarchically porous carbon (N-O-P-HHPC) derived from SJ was firstly prepared by one-step activation and carbonization processes for high performance SCs. The obtained N-O-P-HHPC indicates a higher energy density and long life stability. Therefore, a facile and economic strategy to fabricate N, O and P co-doped HHPC is a considerable candidate for high-performance SCs.

## 2. Experimental section

### Synthesis of N-P-HHPC

The SJ without any impurities was collected from Suzou city in Anhui province and freeze-dried. In brief, a certain mass of KOH was dissolved in deionized water (50 mL), and mixed with 6 g SJ. After soaking for 48 h, the mixed samples were freeze-dried. The mixture samples were heated to 800 °C for 2 h at the rate of 2 °C min^−1^. After cooled down to room temperature, the black mixture samples were soaked into HCl solution (6 mol L^−1^, 50 mL) at 65 °C for 48 h. Then, the obtained materials were washed to PH = 7 and dried at 65 °C for 24 h. In order to distinguish different samples, the as-prepared samples were labeled as N-O-P-HHPC-0, N-O-P-HHPC-1, N-O-P-HHPC-3 and N-O-P-HHPC-6, corresponding to 0 g, 1 g, 3 g and 6 g KOH, respectively. The elemental contents of all samples were measured by using the X-ray photoelectron spectroscope (XPS, Thermo Scientific ESCALab 250Xi system). The detailed characterizations and electrochemical measurements are shown in the ESI.†

## 3. Results and discussion

**Fig. 1:**
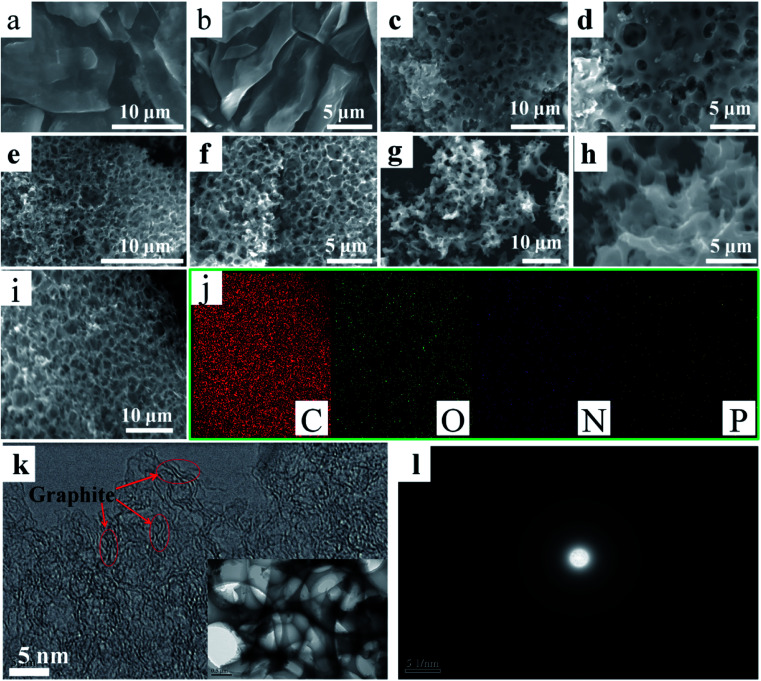
SEM images of N-O-P-HHPC-0 (a and b), N-O-P-HHPC-1 (c and d), N-O-P-HHPC-3 (e and f) and N-O-P-HHPC-6 (g and h), SEM image of N-O-P-HHPC-3 (i), and corresponding elemental mapping images of C, O, N and P (j). HRTEM image (k) and SAED pattern (l) of N-O-P-HHPC-3. Inset of (k) is TEM image of N-O-P-HHPC-3.

The surface microstructures and morphologies of N-O-P-HHPC-0, N-O-P-HHPC-1, N-O-P-HHPC-3 and N-O-P-HHPC-6 were measured by using SEM. Notably, it can be seen that N-O-P-HHPC-0 shows smooth block structure without obviously porous structure as shown in Fig. 1a and b. With the increase mass of KOH, the proportion of micropores increases and the pore density also increases. Therefore, appropriate porous structure can be obtained by controlled the mass of KOH. As shown in Fig. 1c and d, N-O-P-HHPC-1 displays obvious porous structure due to the activating agent of KOH. When the mass value of KOH is 3 g, N-O-P-HHPC-3 appears more porous structure, which looks like honeycomb structure as shown in Fig. 1e and f. With the mass of KOH increases to 6 g, the honeycomb-like porous structure disappears as shown in Fig. 1g and h. The SEM image of N-O-P-HHPC-3 and corresponding elemental mapping images of C, O, N and P are shown in Fig. 1i and j, indicating the uniform C, O, N and P distributions. The porous framework structure of N-O-P-HHPC-3 was further characterized by TEM, HRTEM and SAED. As shown in Fig. 1k, N-O-P-HHPC-3 exhibits 3D interconnected porous structure, which can facilitate ion electrolyte transport and reduce ion diffusion resistance. It is noted that there are some graphitic-like structure could be observed in HRTEM image, which is attributed to the interlayer spacing of graphite (002). In addition, the diffraction ring in the corresponding SAED pattern is blurred, indicating the defected/disordered structure, which can provide more adsorption sites to enhance the capacitance performance.

**Fig. 2:**
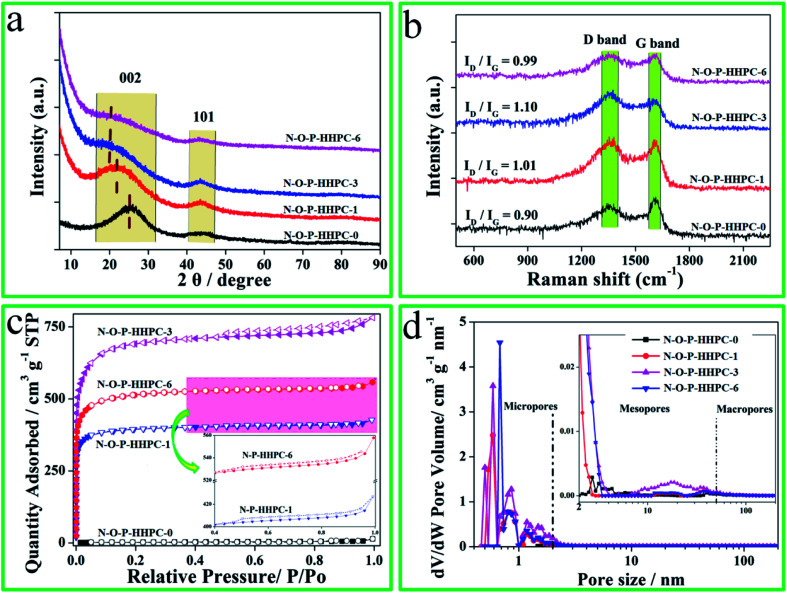
XRD patterns (a), Raman spectra (b), N_2_-adsorption and desorption isotherms (c) and pore size distributions (d) of N-O-P-HHPC-0, N-O-P-HHPC-1, N-O-P-HHPC-3 and N-O-P-HHPC-6.

To further testify the defected/disordered structure of as-prepared samples, XRD patterns and Raman spectra were employed by X-ray diffraction (Rigaku Smartlab) and Raman spectroscopy (LabRAM HR800), and the results are shown in Fig. 2. As-prepared samples exhibit a broad peak at about 23° and a weak peak at 46° as shown in Fig. 2a, corresponding to (002) and (101), which reflects the defected/disordered structure.^39^ Compared with N-O-P-HHPC-0, the broad peak at about 23° of others shift to a lower angle, indicating the defective or disorderly carbon. Moreover, it is clear that the patterns of N-O-P-HHPC-1, N-O-P-HHPC-3 and N-O-P-HHPC-6 at 2*θ* < 10° show an obviously rising tendency, which means the presence of more micropores,^21,27^ resulting in an outstanding specific capacitance. It is consistent with HRTEM results, which favors the improvement of capacitance performance. As shown in Fig. 2b, it is obviously found that the peaks of Raman spectra at about 1350 cm^−1^ and 1580 cm^−1^ are assigned to D band (defective or disordered carbon) and G band (graphitic layers),^39,40^ respectively. As known, as-prepared samples with defected/disordered structure can provide more adsorption sites and the channel for ions to rapidly diffuse and transfer, resulting in the enhanced electrochemical performance. The degree of defective or disordered (*I*_D_/*I*_G_) is presented in Fig. 2b. The *I*_D_/*I*_G_ ratio values of N-O-P-HHPC-0, N-O-P-HHPC-1, N-O-P-HHPC-3 and N-O-P-HHPC-6 are 0.90, 1.01, 1.10 and 0.99, respectively. Obviously, the *I*_D_/*I*_G_ ratio value of N-O-P-HHPC-3 is the largest, indicating the degree of defect or disorder is the largest.^41^

In order to investigate the pore structure more accurately, N_2_-adsorption and desorption measurement was characterized by ASAP 2020 system (Micromeritics, Norcross, GA). The N_2_-adsorption and desorption isotherms are presented in Fig. 2c. Compared with N-O-P-HHPC-0, N_2_-adsorption and desorption isotherms of others exhibit type-I and type-IV isotherm with a strong adsorption (*P*/*P*_0_ < 0.05), broadened knees (0.05 < *P*/*P*_0_ < 0.4) and the hysteresis (*P*/*P*_0_ > 0.5), indicating the existence of micropores and mesopores.^37,42,43^ The N_2_ adsorption amount of N-O-P-HHPC-3 is the largest and the hysteresis is more obviously, which means the existence of more micropores and mesopores. The SSA and pore size distributions of N-O-P-HHPC-0, N-O-P-HHPC-1, N-O-P-HHPC-3 and N-O-P-HHPC-6 were analyzed *via* Brunauer–Emmett–Teller model and DFT method from N_2_-adsorption and desorption isotherms, respectively. The results are shown in Table 1 and Fig. 2d. The N-O-P-HHPC-3 features high surface area (2068.9 m^2^ g^−1^) and the largest pore volume (1.2 m^3^ g^−1^) than those of N-O-P-HHPC-0, N-O-P-HHPC-1 and N-O-P-HHPC-6, which indicates that as-prepared N-O-P-HHPC-3 has a superior rate capability.

**Table 1:** SSA (*S*_BET_, *S*_mic_ and *S*_ext_), *V*_total_, the elemental contents (C, O, N and P) and *R*_ct_ of N-O-P-HHPC-0, N-O-P-HHPC-1, N-O-P-HHPC-3 and N-O-P-HHPC-6

	*S*_BET_ (m^2^ g^−1^)	*S*_mic_ a (m^2^ g^−1^)	*S*_ext_ b (m^2^ g^−1^)	*V*_total_ (cm^3^ g^−1^)	Content^c^ (atomic%)				*R*_ct_ (Ω)
					C	O	N	P	
N-O-P-HHPC-0	6.4	6.1	0.3	0.011	77.9	13.0	7.2	1.6	0.58
N-O-P-HHPC-1	1167.2	1000.3	166.9	0.65	81.2	12.9	4.8	1.0	0.08
N-O-P-HHPC-3	2068.9	1592	476.9	1.2	89.8	8.4	1.5	0.4	0.05
N-O-P-HHPC-6	1533.9	1221.9	312	0.83	88.5	11.3	0.2	0	0.06

a Specific surface area of micropores.

b Specific surface area of other pores.

c The elemental contents of samples were employed by XPS.

**Fig. 3:**
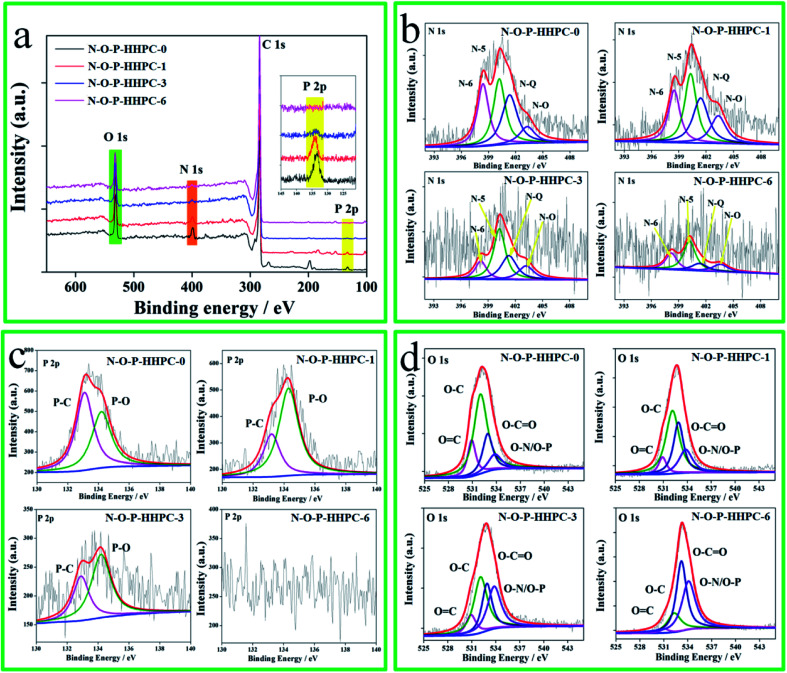
XPS survey spectra (a), N 1s spectra (b), P 2p spectra (c) and O 1s spectra (d) of N-O-P-HHPC-0, N-O-P-HHPC-1, N-O-P-HHPC-3 and N-O-P-HHPC-6.

In order to better prove the element composition of as-prepared samples, XPS measurements were characterized by Thermo Scientific ESCALab 250Xi system. As shown in Fig. 3, there are four mainly peaks at about 533, 400, 284 and 135 eV, corresponding to the binding energies of O 1s, N 1s, C 1s and P 2p, indicating N, O and P self-doping during carbonizing.^17,22,30^ According to the N, O and P contents of all samples (Table 1), it is obvious found that N, O and P are consumed during carbonization and activation process. The N, O and P contents of N-O-P-HHPC-3 are 1.5, 8.4 and 0.4 atomic%, respectively. The Fig. 3b–d displays the N 1s, P 2p and O 1s of all samples, respectively. The N 1s of all samples are fitted and shown in Fig. 3b, which indicates that the N element mainly includes pyridinic-N-oxide (403.3 eV, N-O) quaternary-N (401.5 eV, N-Q), pyrrolic/pyridone-N (400.1 eV, N-5) and pyridinic-N (398.4 eV, N-6).^27,30^ According to previous report, the N-5 and N-6 atoms can mainly contribute pseudocapacitance to enhance the specific capacitance.^44^ Meanwhile, N-Q and N-O can enhance the electron transfer and hydrophilia,^33^ respectively. The P 2p can be fitted into two peaks at 133.2 eV and 134.3 eV, corresponding to P–C and P–O, respectively, which can enhance hydrophilia.^44,45^ Obviously, the P element of N-O-P-HHPC-6 is disappeared due to the etching effect of KOH, which improves its contact resistance.^30^ The O element can be fitted to four peaks at 530.9 eV, 532.1 eV 532.9 eV and 533.8 eV, corresponding to O=C, O–C, O–C=O and O–N/O–P, respectively, which can enhance the hydrophilia and some capacitive performance.^43,45^ These results of XPS further confirm that N, O and P can be self-doped into N-O-P-HHPC, resulting in the improved electrochemical performance.

**Fig. 4:**
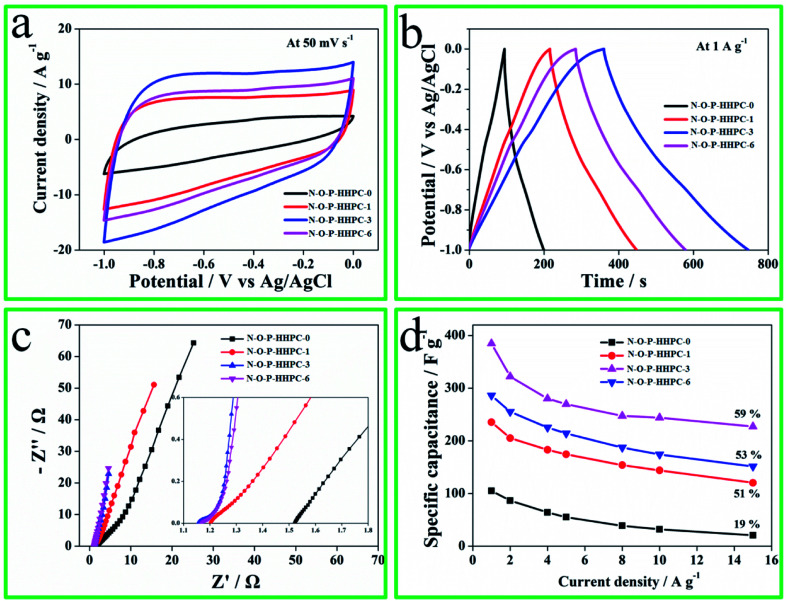
The electrochemical performances of N-O-P-HHPC-0, N-O-P-HHPC-1, N-O-P-HHPC-3 and N-O-P-HHPC-6 tested in 6 KOH using the three electrode system. (a) CV curves at 50 mV s^−1^. (b) GCD curves at 1 A g^−1^. (c) EIS curves, the inset at lower right corner is the zoom-in Nyquist plots. (d) The specific capacitances at different current densities.

In the three electrode system, the electrochemical performances (CV, EIS and GCD) of N-O-P-HHPC-0, N-O-P-HHPC-1, N-O-P-HHPC-3 and N-O-P-HHPC-6 were tested in 6 M KOH, and the results are shown in Fig. 4, S1 and S2.† As shown in Fig. S1,† all CV curves without any redox peak show approximately symmetrical rectangle. The area of CV curve corresponds to the specific capacitance. The larger area of CV curve at the same scan rate indicates the larger specific capacitance. From the Fig. 4a, CV curve of N-O-P-HHPC-3 displays the largest area than others, indicating the largest specific capacitance. Meanwhile, the GCD curve of N-O-P-HHPC-3 exhibits the longest discharge time, also meaning the largest specific capacitance, which is consistent with the results of CV curves. The largest specific capacitance of N-O-P-HHPC-3 is 386 F g^−1^ at 1 A g^−1^, because the numerous micropores and mesopores provide more adsorption sites and N, O and P co-doping can produce pseudocapacitance. EIS curves of N-O-P-HHPC-0, N-O-P-HHPC-1, N-O-P-HHPC-3 and N-O-P-HHPC-6 are displayed in Fig. 4c. It is obviously seen that N-O-P-HHPC-3 exhibits more vertical slope line in the low frequency region, indicating more capacitance performance. In the high frequency region, the charge transfer resistance (*R*_ct_ = 0.05 Ω) of N-O-P-HHPC-3 displays the smallest in Table 1, due to its interconnected porous structure. At the same time, the curves of specific capacitance *versus* current density (as shown in Fig. 4d) for the N-O-P-HHPC-3 also shows the highest specific capacitance. Moreover, the capacitance retention of N-O-P-HHPC-3 is 59% at 15 A g^−1^, which is higher than N-O-P-HHPC-1 (51%) and N-O-P-HHPC-6 (53%), due to its larges specific surface area with 3D hierarchically porous structure (micropores, mesopores and macropores). The existence of numerous micropores can provide ion adsorption sites, and the mesoporous and macroporous structures can form ion fast channels, resulting in enhanced electrochemical performances.

**Fig. 5:**
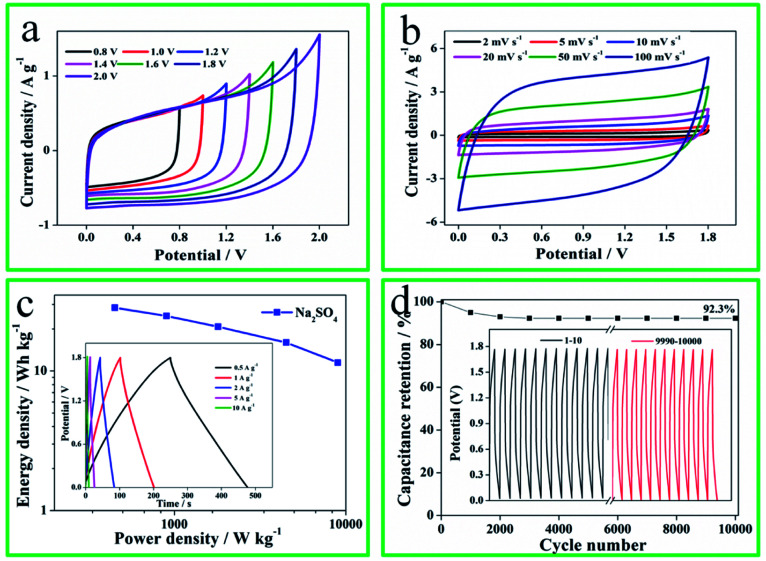
The electrochemical performances of N-O-P-HHPC-3//N-O-P-HHPC-3 symmetrical SCs using 1 M Na_2_SO_4_ gel electrolyte. (a) CV curves of different potential windows at 10 mV s^−1^. (b) CV curves of different scan rates. (c) Ragone plot, inset of (c) is GCD curves at different current densities. (d) Cycling stability efficiency at 10 A g^−1^, inset of (d) is the first 10 cycles and the last 10 cycles.

To further reveal the capacitance performances, the symmetrical SCs was constructed by N-O-P-HHPC-3 electrode and tested using 1 M Na_2_SO_4_ gel electrolyte by two electrodes system, and the results are shown in Fig. 5. Notable, the potential window of the N-O-P-HHPC-3‖N-O-P-HHPC-3 symmetrical SCs can reach up to 1.8 V (Fig. 5a). The CV curves of the N-O-P-HHPC-3‖N-O-P-HHPC-3 symmetrical SCs at different scan rates (2–100 mV s^−1^) display approximately symmetric rectangle in Fig. 5b, owning to its hierarchically porous structure that provides more adsorption sites and the channel for ions to rapidly diffuse and transfer. Meanwhile, GCD curves at 0.5 A g^−1^ show equicrural triangle with a small internal resistance (IR) drop. The Ragone plot of the N-O-P-HHPC-3//N-O-P-HHPC-3 symmetrical SCs is shown in Fig. 5c. When the power density is 449.9 W kg^−1^, the energy density can be reach up to 28.4 W h kg^−1^, which is higher than the reported energy density of biomass carbon based supercapacitors (Table S1†). When the power density increases to 8992.2 W kg^−1^, the energy density of 11.5 W h kg^−1^ can still be delivered. Meanwhile, the cycling stability of the symmetrical SCs with the potential window of 1.8 V is analyzed at 10 A g^−1^ for 10 000 cycles, exhibiting an outstanding long life cycle (capacitance retention of 92.3%) in Fig. 5d.

**Fig. 6:**
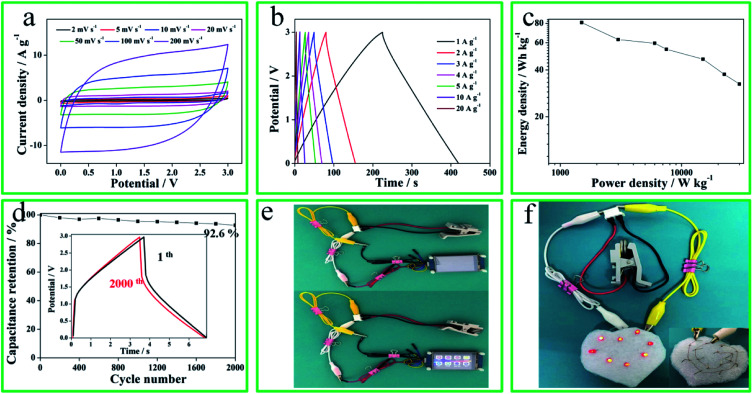
The electrochemical performances of N-O-P-HHPC-3//N-O-P-HHPC-3 symmetrical SCs using EMIMBF4 as electrolyte. (a) CV curves at 2–200 mV s^−1^. (b) GCD curves at 1–20 A g^−1^. (c) Ragone plot. (d) The capacitance retention *vs.* cycle numbers. The inset of GCD curves at 1st and 2000th. (e) Digital display lighted by three N-O-P-HHPC-3//N-O-P-HHPC-3 symmetrical SCs devices connected in series. (f) The eight red LED bulbs with heart shape powered by two coin-type symmetrical SCs connected in series.

To further investigate the superior capacitive performances of N-O-P-HHPC-3, a two-electrode coin-type symmetrical SCs was assembled with EMIMBF4 as electrolyte. As shown in Fig. 6a and b, CV curves display rectangular shapes and GCD plots exhibit perfect linear and symmetrical shapes, indicating ideal EDLC behavior. Meanwhile, the potential window of the coin-type symmetrical SCs can reach to 3 V, which is better to enhance the energy density. The Ragone plot of the coin-type symmetrical SCs is shown in Fig. 6c. When the power density increases, the energy density decreases. The ultrahigh energy density is 80.8 W h kg^−1^ at 1500 W kg^−1^, which is larger than previous literature reports (Table S1†). The cycling stability of the coin-type symmetrical SCs was analyzed, and the results are shown in Fig. 6d. The two-electrode coin-type symmetrical SCs demonstrates an excellent long life cycles. The capacitance retention reaches up to 92.6% after 2000 cycles (GCD curves) at 20 A g^−1^. Benefiting from the integrated high energy and power properties, two coin-type symmetrical SCs in series can power a small display screen (5 V, Fig. 6e) and eight red LED modules (Fig. 6f), indicating its great potential for high-performance SCs application.

## 4. Conclusion

In summary, the N, O and P co-doped honeycomb-like hierarchically porous carbon was successfully prepared from *Sophora japonica* by a facile, relatively green and efficient strategy. The specific capacitance of the optimized N-O-P-HHPC-3 can reaches up to 386 F g^−1^ at current density of 1 A g^−1^ with the capacitance retention of 59%. The excellent performance of N-O-P-HHPC-3 is assigned to the contribution of the largest specific surface area of 2068.9 m^2^ g^−1^ with interconnect honeycomb-like hierarchical porous structure (micropores, mesopores and macropores) and N (1.5 atomic%), O (8.4 atomic%) P (0.4 atomic%) co-doping. Meanwhile, the 1.8 V N-O-P-HHPC-3//N-O-P-HHPC-3 symmetrical SCs using high-voltage 1 M Na_2_SO_4_ gel electrolyte shows an outstanding cycling stability (7.7% capacitance loss after 10 000 charge and discharge cycles). Furthermore, the two-electrode coin-type symmetrical SCs exhibits an ultrahigh the energy density of 80.8 W h kg^−1^ at 1500 W kg^−1^ using EMIMBF4 as electrolyte. When the two coin-type symmetrical SCs are connected in series, the eight red LED in parallels and the small display screen can be powered, which directly demonstrates tremendous opportunities to be applied in high performance energy storage.

## Conflicts of interest

There are no conflicts to declare.

## Supplementary Material

RA-009-C9RA06934H-s001
