# Supplementary material for: N, O and P co-doped honeycomb-like hierarchical porous carbon derived from Sophora japonica for high performance supercapacitors
Source: RSC Adv. 2019 Nov 13;9(64):37171–8. doi: 10.1039/c9ra06934h (PMC9075545; doi:10.1039/c9ra06934h)
Supplement: RA-009-C9RA06934H-s001 [file RA-009-C9RA06934H-s001.pdf]

Electronic Supplementary Material (ESI) for RSC Advances.
This journal is © The Royal Society of Chemistry 2019

Supporting Information

N, O and P Co-doped Honeycomb-like Hierarchical Porous Carbon Derived from *Sophora Japonica* for High Performance Supercapacitors

Li zhang, Yuxia Zhu, Guangzhen Zhao, Yanjiang Li, Guang Zhu*

Key Laboratory of Spin Electron and Nanomaterials of Anhui Higher Education Institutes, Suzhou University, Suzhou 234000, PR China

*Corresponding author, E-mail: guangzhu@ahszu.edu.cn

1. Characterization

The surface morphologies and microstructures of all samples were observed by transmission electron microscopy (TEM, Tecnai G2 F20), high resolution transmission electron microscopy (HRTEM), selected area electron diffraction (SAED) and scanning electron microscopy (SEM, SU1510). The crystallinity of the samples was characterized by Raman spectroscopy (Horiba Horiba Jobin Yvon LabRAM HR800) and X-ray diffraction (XRD, Rigaku Smartlab). The chemical bond structure and elemental analysis of materials were examined by the X-ray photoelectron spectroscope (XPS) using Thermo Scientific ESCALab 250Xi system. The N_2 adsorption/desorption isotherms of as-prepared samples were obtained by ASAP 2020 system (Micrometitics, Norcross, GA). The Brunauer-Emett-Teller (BET) specific surface areas (SSA), total pore volumes (V_{total}) and pore size distributions were analyzed through N_2 adsorption/desorption isotherms.

2. Electrochemial measurements

In a three-electrode system, the electrochemical performances of as-prepared samples were measured by electrochemical workstation (Metrohm, AUTOLAB). The obtained slurry was fabricated by mixing the obtained electrode sample (80%), polyvinylidene fluoride (PVDF, 10%) and acetylene black (10%) using a certain amount of N-methyl-2-pyrrolidone (NMP) as solvent, which was cast on a carbon paper (20 × 35 mm^2) and dried (80 ˚C for 12 h) as working electrode. Ag/AgCl electrode and a Pt foil electrode (20 × 20 mm^2) were used as reference and counter electrode, respectively. The electrochemical impedance spectroscopy at an open circuit potential with amplitude of 5 mV in the frequency range of 0.01-100000 Hz (EIS), cyclic voltammetry (CV), and galvanostatic charge-discharge (GCD) were

measured using a 6 M KOH aqueous solution. The specific capacitances (C, F g^{-1}) of all samples were obtained from the GCD curves as follows:

$$C = \frac{I_d \times \Delta t}{m \times \Delta V} \quad (1)$$

where ΔV (V), I_d (A), Δt (s) and m (g) is the applied voltage window, the discharge current, discharge time and the mass of active materials.

The symmetrical SC was assembled by two N-P-HHPC-3 electrodes and measured using a two electrode system. CV, GCD and cycle life measurements (CHI660E, Chenhua Instruments Co. Ltd., Shanghai) were tested in 1 M Na_2SO_4 gel electrolyte and EMIMBF4 solution, respectively. The specific capacitance (C, F g^{-1}), energy density (E, Wh kg^{-1}) and power density (P, W kg^{-1}) of symmetrical SC can be calculated by the GCD curves as following equations:

$$E = \frac{1}{7.2} \times C \times \Delta V^2 \quad (2)$$

$$P = 3600 \times \frac{E}{\Delta t} \quad (3)$$

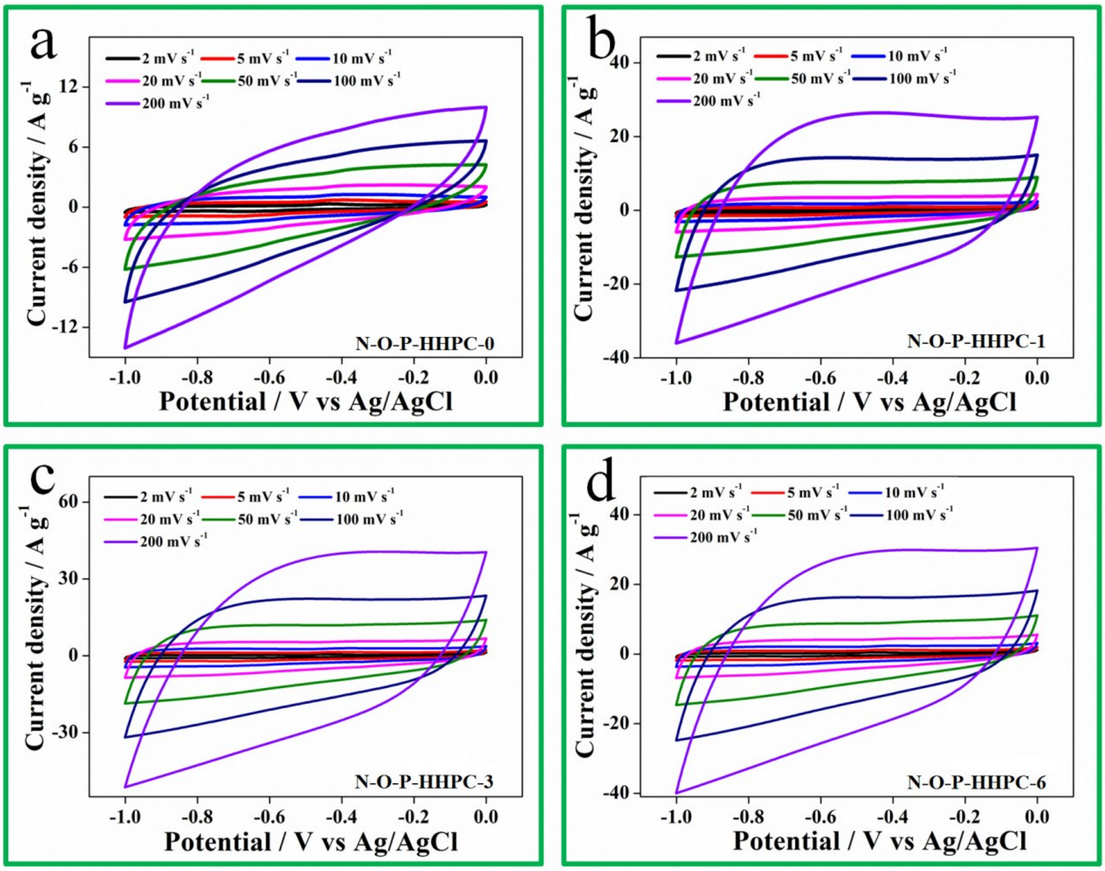

Figure S1 CV curves at different scan rates of N-O-P-HHPC-0 (a), N-O-P-HHPC-1 (b), N-O-P-HHPC-3 (c) and N-O-P-HHPC-6 (d).

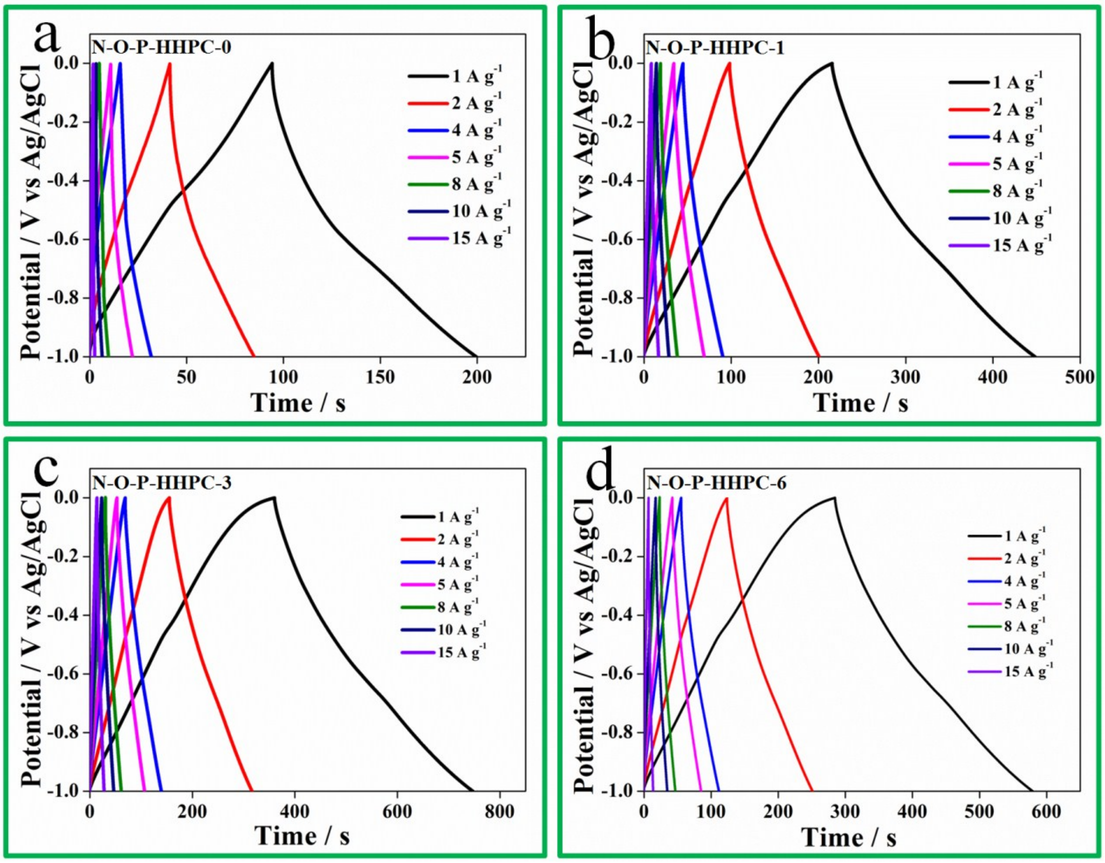

Figure S2 GCD curves at different current densities of N-O-P-HHPC-0 (a), N-O-P-HHPC-1 (b), N-O-P-HHPC-3 (c) and N-O-P-HHPC-6 (d).

Table S1 Electrochemical performances of biomass based carbon material

Biomass precursor	SSA (m^2 g^{-1})	C (F g^{-1}) / Current density (A g^{-1})	E (W h kg^{-1})/P (W kg^{-1})	Ref.
Sugar	1005	250/1	8.7/500 (6 M KOH)	[1]
Willow catkin	1533	298/0.5	21/180 (1M Na_2SO_4)	[2]
Perilla frutescens	*655*	270/0.5	14.8/490 (1M Na_2SO_4)	[3]
Weed	1484	391/0.5	20.15/500 (1M Na_2SO_4)	[4]
Chestnut shell	691.8	402.8/0.5	26.6/445.5 (1M Na_2SO_4)	[5]
Baobab fruit shells	1059.3	233.48/1	20.86/400 (1M Na_2SO_4)	[6]
Tobacco rods	1886	286.6/1	31.3/500(1M Et4NBF4)	[7]
Coal tar	1517	274/0.05	--	[8]
Sunflower stalk	1505	365/1	35.7/989 (Et4NBF4)	[9]
Yogurt	1300	225/2	7/5000 (6 M KOH)	[10]
Boat-fruited sterculia seeds	3243	--	22.4/500 (1M Na_2SO_4)	[11]
Tremella	1097	299.3/0.5	42.4/760 (Et4NBF4)	[12]
Petroleum asphalt	3581	277/0.05	14.2/445 (1M Na_2SO_4)	[13]
Straw cellulose waste	2297	312.6/0.5	28.6/14090 (Et4NBF4)	[14]
Broad bean shells	655.4	202/0.5	--	[15]
Chitosan	1129.6	316/0.2	--	[16]
Bamboo char	1732	222/0.5	6.7/100 (6 M KOH)	[17]
Soybean	1749	243.2/0.5	12.5/450 (0.5 M Na_2SO_4)	[18]
Corn straw	1771	222/1	--	[19]
Shrimp shells	1946	322/0.5	11.2/110 (6 M KOH)	[20]
**Sophora Japonica**	**2068.9**	**386/1**	**28.4/449.9 (1M Na_2SO_4)**<br>**80.8/1500 (EMIMBF4)**	**Our work**

REFERENCES

[1] F. Xu, Z. Tang, S. Huang, L. Chen, Y. Liang, W. Mai, *Nat. Commun.*, 2015, **6**, 7221.

[2] Y. Li, G. Wang, T. Wei, Z. Fan, P. Yan, *Nano Energy*, 2016, **19**, 165-175.

[3] B. Liu, Y. Liu, H. Chen, M. Yang, H. Li, *J. Power Sources*, 2017, **341**, 309-317.

[4] W. Zhou, S. Lei, S. Sun, X. Ou, Q. Fu, Y. Xu, *J. Power Sources*, 2018, **402**, 203-212.

[5] L. Wan, X. Li, N. Li, M. Xie, C. Du, Y. Zhang, *J. Alloy. Compd.*, 2019, **790**, 760-771.

[6] A. A. Mohammed, C. Chen, Z. Zhu, *J. Colloid Interf. Sci.*, 2019, **538**, 308-319.

[7] Y. Q. Zhao, M. Lu, P. Y. Tao, Y.J. Zhang, X. T. Gong, Z. Yang, *J. Power Sources*, 2016, **307**, 391-400.

[8] X. Xie, X. He, X. Shao, S. Dong, N. Xiao, J. Qiu, *Electrochimica Acta*, 2017, **246**, 634-642.

[9] X. Wang, S. Yun, W. Fang, C. Zhang, X. Liang, Z. Lei, *ACS Sustainable Chem. Eng.*, 2018, **6(9)**, 11397-11407.

[10] M. Wahid, G. Parte, D. Phase, S. Ogale, *J. Mater. Chem. A*, 2015, **3(3)**, 1208-1215.

[11] Y. Ding, L. Mo, C. Gao, X. Liu, T. Yu, W. Chen, *ACS Sustainable Chem. Eng.*, 2018, **6(8)**, 9822-9830.

[12] D. Chen, L. Li, Y. Xi, J. Li, M. Lu, J. Cao, *Electrochimica Acta*, 2018, **286**, 264-270.

[13] L. Pan, Y. Wang, H. Hu, X. Li, J. Liu, L. Guan, *Carbon*, 2018, **134**, 345-353.

[14] Z. Chen, X. Wang, B. Xue, Q. Wei, L. Hu, Z. Wang, *ChemSusChem*, 2019, **12(7)**, 1390-1400.

[15] G. Xu, J. Han, B. Ding, P. Nie, J. Pan, H. Dou, *Green Chem.*, 2015, **17**, 1668-1674.

[16] Z. Lin, X. Xiang, S. Peng, X. Jiang, L. Hou, *J. Electroanal. Chem.*, 2018, **823**, 563-572.

[17] Y. Gong, D. Li, C. Luo, Q. Fu, C. Pan, *Green Chem*., 2017, **19(17)**, 4132-4140.

[18] G. Lin, R. Ma, Y. Zhou, Q. Liu, X. Dong, J. Wang, *Electrochimica Acta*, 2018, **261**, 49-57.

[19] Y. Lu, S. Zhang, J. Yin, C. Bai, J. Zhang, Y. Li, *Carbon*, 2017, **124**, 64-71.

[20] W. Tian, Q. M. Gao, L. M. Zhang, C. Yang, Z. Y. Li, Y. Tan, W. Qian, H. Zhang, *J. Mater. Chem. A*, 2016, **4**, 8690-8699.
